# Wideband Versatile Receiver for CubeSat Microwave Front-Ends

**DOI:** 10.3390/s22229004

**Published:** 2022-11-21

**Authors:** Emanuele Cardillo, Renato Cananzi, Paolo Vita

**Affiliations:** 1 Department of Engineering, University of Messina, 98166 Messina, Italy; 2 Italspazio S.r.l., 95037 Catania, Italy

**Keywords:** CubeSat, down converter, microwaves receivers, satellite communications, space applications, spurious reduction, tuners, internet of things, wide bandwidth

## Abstract

One of the main features of CubeSats is represented by their extreme versatility, e.g., maintaining the same overall structure for different purposes. This requires high technological flexibility achievable in a cost-effective way while maintaining compact sizes. In this contribution, a microwave receiver specifically designed for CubeSat applications is proposed. Due to the wide input operating bandwidth, i.e., 2 GHz–18 GHz, it can be exploited for different purposes, e.g., satellite communication, radars, and electronic warfare systems. This is beneficial for CubeSat systems, whereby the possibility to share the same front-end circuit for different purposes is a key feature in reducing the overall size and weight. The downconverter was designed to minimize the spurious contributions at low frequency by taking advantage, at the same time, of commercial off-the-shelf components due to their cost-effectiveness. The idea behind this work is to add flexibility to the CubeSat communication systems in order to be reusable in different contexts. This feature enables new applications but also provides the largest bandwidth if required from the ground system. An accurate experimental characterization was performed to validate the downconverter performance with the aim of allowing easy system integration for the new frontier of CubeSat technologies. This paves the way for the most effective implementation of the Internet of Things (IoT), machine-to-machine (M2M) communications, and smart-everything services.

## 1. Introduction

Since the first CubeSat launch in 2003, approximately 2.000 CubeSats have been sent into outer space [1,2]. The exponential growth of the number of CubeSats can be attributed to the great interest of both space agencies and private companies. Above all, the research activities of academic teams are fueled by the cost-effectiveness of CubeSats compared to bulkier satellites [3]. Moreover, the availability of commercial off-the-shelf (COTS) components, easily accessible with a limited budget, fueled the research activities in the space sector.

CubeSats are compact and lightweight nanosatellites characterized by sizes complying with the standard dimensions of 10 cm × 10 cm × 10 cm for a single unit. The Cube represents a standard unit (U-), which must weigh less than 1.33 Kg. A maximum number of 24 units can be assembled to obtain complex structures with advanced performance [4]. The requirement of complying with standard dimensions must not be intended as a limitation. Indeed, the designer can take the opportunity to send a CubeSat into outer space by joining existing launch opportunities, where a dedicated section is available to carry nanosatellites of standard dimensions. This aspect contributes to keeping down the launch costs.

In a satellite communication system, one of the most critical stages is represented by the receiver. This is a consequence of the changing landscape of applications and technologies based on satellites developed in recent years [5]. Nowadays, a whole set of applications are based on concepts such as the Internet of Things (IoT), machine-to-machine (M2M) communications and smart-everything services, e.g., the possibility to provide connection capabilities in remote locations. As an example, by equipping a CubeSat constellation with IoT communication systems, it would be possible to establish a connection with IoT ground-segment sensors placed in remote locations, otherwise inaccessible from ground communication systems.

Developing such a service requires more bandwidth to be available for the user [6,7,8]. In this scenario, designing wide bandwidth receivers represents a critical hardware requirement for satellite-based approaches.

In the scientific literature, many research activities focused on a manifold set of topics concerning CubeSat receivers can be found [9,10,11,12,13,14,15,16,17,18,19,20,21,22,23,24,25,26]. The most relevant related contributions are reported and briefly described in Section 2.

It is worth noting that these works show only operating bandwidths narrower than the 2–18 GHz bandwidth of this contribution. This allows the authors to highlight the main technological limitations of the current receivers, represented by the difficulties in obtaining wide working bandwidths by maintaining low spurious levels without significantly increasing the cost of the system.

To fill this gap, in this contribution, a microwave down-converter is proposed as a multi-purpose CubeSat receiving stage. The performance of the circuit was first simulated within the Cadence AWR Microwave Office design environment software in order to accurately select the required components and tune the final system behavior. The complete receiving chain has been designed with the aim of creating a compact system easily integrable into nanosatellite front-ends. Particular attention has been paid to keeping down the costs by using COTS components. The proposed topology uses a dual-conversion architecture exploiting two local oscillators (LOs) stages to provide a severe reduction of both the number and level of spurious in the operating band. Indeed, the first up-conversion stage allows us to filter the signal at a higher frequency, whereby the filtering task can be accomplished more effectively, i.e., with a better spurious rejection [27]. Finally, the signal was down-converted to the baseband in order to be processed more comfortably.

The wide bandwidth, i.e., 2–18 GHz, and the employed components make the system very interesting for a great number of applications, e.g., from satellite communication to radar front-ends [28,29,30,31,32]. The paper is organized as follows. The main works related to this topic have been depicted in Section 2. The operating principle, simulations, and characterization of the components of the chain have been reported in Section 3. In Section 4, the performance of the receiver was shown. Finally, the conclusions are drawn in Section 5.

## 2. Related Works

The recent research activities concerning CubeSats are focused on a wide set of topics, e.g., the development of compact and high gain antennas, either stationary or composed of deployable structures, and able to increase the radiation area after reaching the correct CubeSat position and attitude with respect to the Earth [9,10,11,12,13]. Many valuable contributions are instead focused on technological aspects related to the CubeSat position and attitude. In [14], the NASA/NORAD two-line element (TLE) position and velocity calculation were validated by means of an on-board Global Positioning System (GPS) receiver, whereas in [15], the attitude of the UltraViolet & infrared Sensors at high Quantum efficiency was determined by means of a tri-axial attitude determination (TRIAD) method and then improved by means of an extended Kalman filter (MEKF). Recent works have also focused on the proper determination of the power link budget, on the improvement of the efficiency of solar panels and on the enhancement of the energy accumulation systems performance [16,17].

In the scientific literature, the topic of designing premium performance versatile receivers is of great interest, although a very wide frequency microwave downconverter has still not been addressed. The availability of wide-bandwidth downconverters would have great potential in a huge number of applications, e.g., satellite communication, radars, etc., where they might be used in the receiving stages [18,19,20,21,22,23].

As an example, in [24], both the down-converting and up-converting sections were designed for GPS applications, with optimum performance in terms of output power and low-level path losses. However, the downconverter was specifically designed for GPS systems, thus with a very narrow operating bandwidth centered at the frequencies of 433 MHz and 1575.42 MHz (L1 band). In [25], a CubeSat transceiver designed, realized and tested to work in the S-band (in detail from 2.025 GHz to 2.29 GHz) with the purpose of transmitting images and videos between a satellite and a ground station was studied. Particular attention was devoted to antennas and filters.

In [26], a prototype radio frequency (RF) receiver for Cubesats was designed and fabricated. Even though it was realized by exploiting COTS components, it was designed to operate within a very narrow bandwidth, i.e., 2025–2110 MHz. However, the characterization was particularly deserving since it was performed both with a board-level approach concerning the verification of the functional requirements and with a component-level approach to accurately characterize each specific component.

This literature review highlights the lack of wideband receivers in the scientific literature, justifying the efforts of the authors in the present study.

## 3. Receiver Design, Simulation and Components Characterization

Designing high-performance CubeSat receivers equipped with double frequency conversion stages for higher spurious rejection was a challenge until a few years ago. This was mainly due to the lack of suitable components, especially for the high-frequency filtering section, which was limited in terms of working bandwidth, rejection level and cost.

The first mixer in the receiving chain was specifically designed to up-convert the input signal to a higher frequency, then the second mixer down-converts again the signal to a lower frequency, namely the intermediate frequency (IF). Even though this circuital topology increases the complexity of the receiver and adds a certain number of components, it offers different advantages, e.g., it is beneficial where high spurious rejection is required. Another important point to consider is the presence of the so-called image frequency. This occurs when an additional tone, i.e., additional with respect to the signal of interest, enters the mixer and generates an output at the same IF frequency as the desired one. This makes the detection of the main signal very challenging. This issue can be mitigated by filtering the signal at the input of the chain, but considering wide input bandwidths, such as the 16 GHz bandwidth of the present contribution, it might be nearly impossible to find a suitable variable microwave filter that can be placed at the input of the chain. By introducing a first up-conversion stage, it is possible to increase the frequency separation between the signal of interest and the other frequency components, thus facilitating the task of filtering unwanted tones.

**Figure 1:**
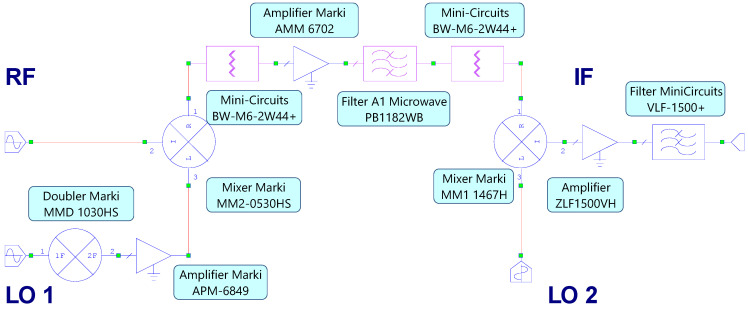
Schematic of the proposed microwave receiver.

The block diagram of the proposed receiver, designed within the Cadence AWR Microwave Office design environment, is reported in Figure 1.

Every component was accurately simulated and tested. Indeed, the components’ losses, if not adequately compensated by means of amplification stages, might under-drive the LO inputs of the different mixers, thus generating undesired behaviors. On the other hand, the dynamic range of the amplifiers must be taken into account to avoid the compression of their gain.

The input signal, after being received by the antenna and amplified, is the MM2-0530HS mixer by Marki Microwave, Inc., Morgan Hill, CA, USA. This block upconverts the signal up to the frequency of 29 GHz. To make possible the up-conversion of the 2–18 GHz input signal, an LO signal spanning from 27 GHz to 11 GHz was required to exploit the sum frequency components at the output of the mixer.

**Figure 2:**
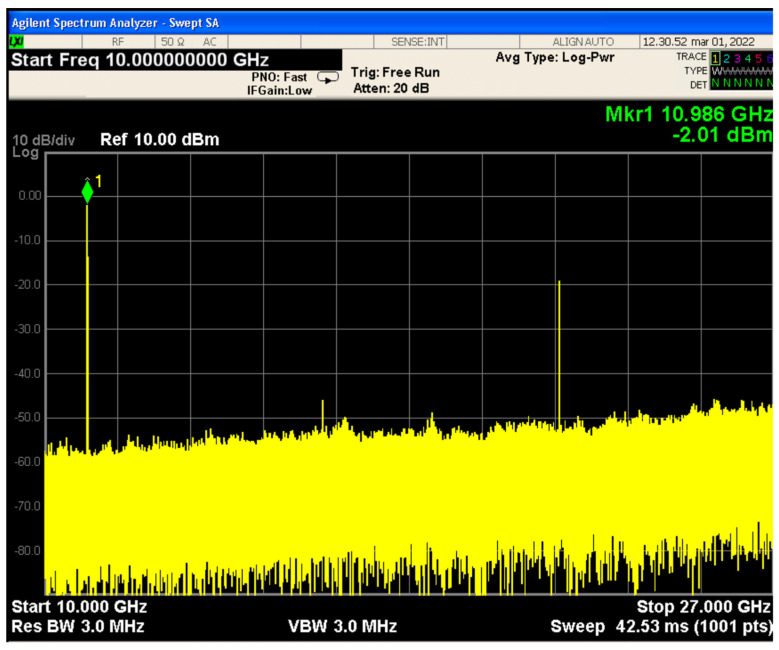
Frequency spectrum at the output of the frequency doubler MMD 1030HS. The input signal was centered at 5.5 GHz.

To lower the costs required to generate such a high-frequency signal, a 13.5–5.5 GHz signal was injected in a frequency doubler stage by exploiting the MMD 1030HS by Marki Microwave, Inc. Multiple multiplication stages could have been employed, but this solution would have been dramatically increased the number of spurious. The proper operation of the frequency doubler was tested by verifying its performance over all the bandwidth. For the sake of brevity, only the output spectrum was measured with an Agilent MXA N9020A spectrum analyzer and by injecting a 5.5. GHz signal at the input of the component is reported. The output spectrum was reported in Figure 2. By taking into account the losses of the employed cables, the overall attenuation of the frequency doubler was measured as equal to 11 dB.

**Figure 3:**
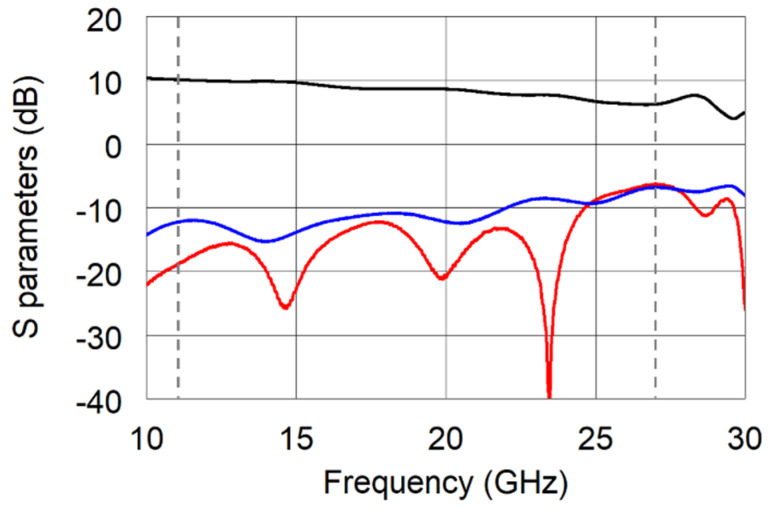
Measured S_21_ (black solid line), S_11_ (blue solid line), and S_22_ (red solid line) of the APM-6849 amplifier.

The first stage mixer requires an LO signal level at least equal to 15 dBm. Considering the doubler conversion loss of around 11 dB, an LO amplifier stage was employed. In detail, the APM-6849 by Marki Microwave, Inc. was chosen for its low-phase noise. The scattering parameters of the amplifier were measured by means of the vector network analyzer E8364A by Agilent Technologies (45 MHz–50 GHz; Figure 3) in order to verify the component performance and tune the system accordingly.

**Figure 4:**
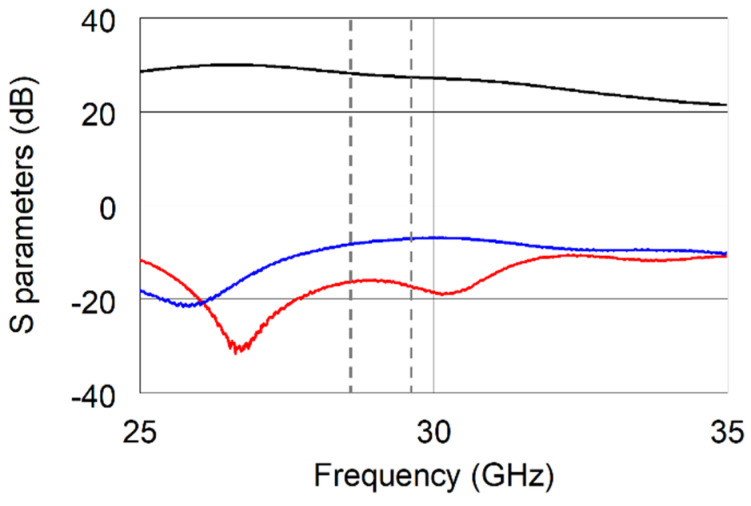
Measured S_21_ (black solid line), S_11_ (blue solid line), and S_22_ (red solid line) of the APM-6702 amplifier.

The up-converted signal was amplified by means of the AMM 6702 amplifier by Marki Microwave, Inc., in order to compensate for the first mixer stage losses. The measured Scattering parameters are reported in Figure 4, where it is worth noting that all the parameters fully comply with the specifications reported in the datasheet.

**Figure 5:**
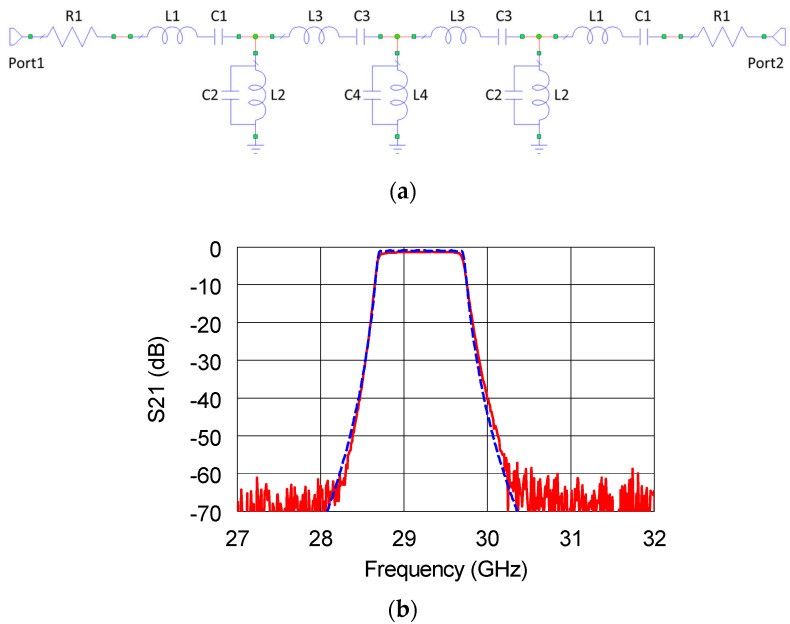
(**a**) Schematic and (**b**) insertion loss of the measured (red solid line) and simulated (blue dashed line) S_21_ for the PB1182WB filter.

Moreover, different fixed attenuator stages were employed to better equalize the system power budget. In order to limit the bandwidth to 1 GHz, thus properly fulfilling the goal of spurious rejection, a PB1182WB bandpass filter by A1 Microwave Ltd. was employed. This is a critical component of the chain, and unfortunately, Microwave Ltd. did not provide the related scattering (S-) parameters. Therefore, the filter performance was modeled by designing an equivalent circuital network. To this purpose, a 7-elements Chebyshev lumped-element topology was designed. In Figure 5a, the schematic of the filter model is shown.

Thereafter, the filter was tested in order to validate the extracted model. The good matching between the real and reproduced curves might be verified by observing Figure 5b, where the measured S_21_ is reported along with the simulated one. The values of the filter elements are reported in Table 1.

**Figure 6:**
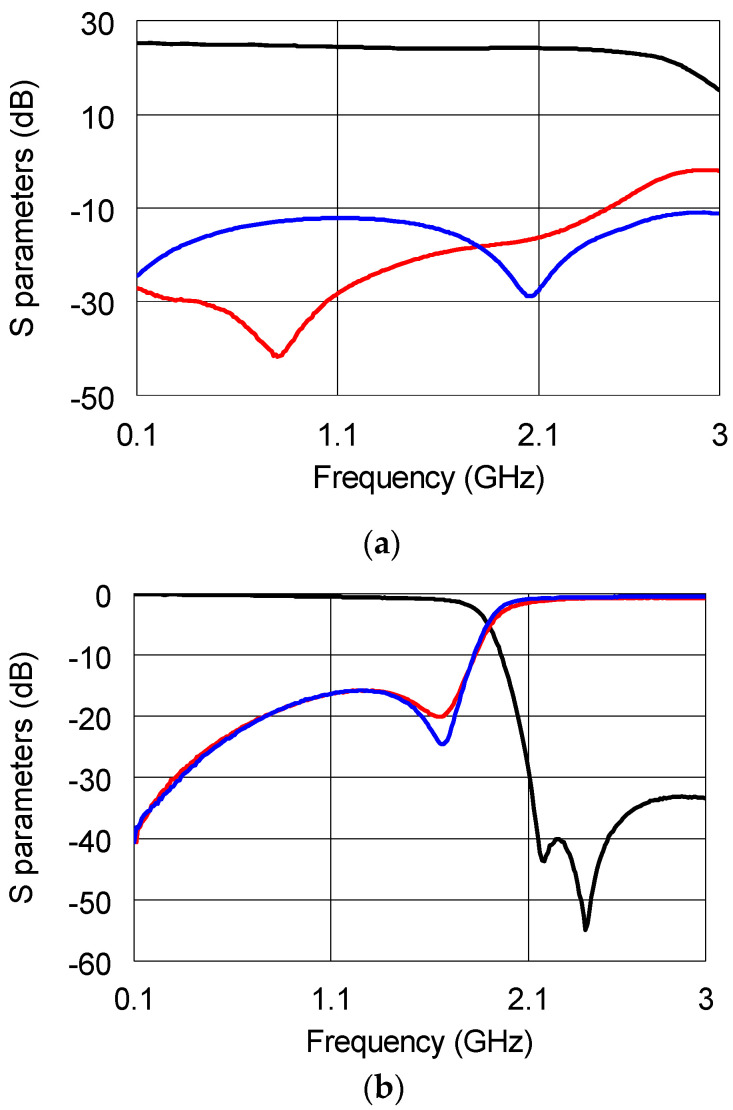
Measured S_21_ (black solid line), S_11_ (blue solid line), and S_22_ (red solid line) of the (**a**) ZFL-1500VH+ amplifier and (**b**) VLF-1500+ filter.

Thereafter, the MM1-1467H mixer was exploited to down-convert the signal down to the frequency of 1 GHz in the down-conversion stage. A 28 GHz signal, synchronized with the LO of the first mixer, was injected into the second LO input. Such a LO value was selected to obtain the central frequency of 1 GHz at the mixer output. The signal is finally amplified and filtered by means of the ZFL-1500VH+ and the VLF-1500+ by MiniCircuits, respectively. The S-parameters of both the amplifier and filter are reported in Figure 6a,b.

At this stage, the IF signal can be easily processed by employing currently available analog-to-digital converters.

**Figure 7:**
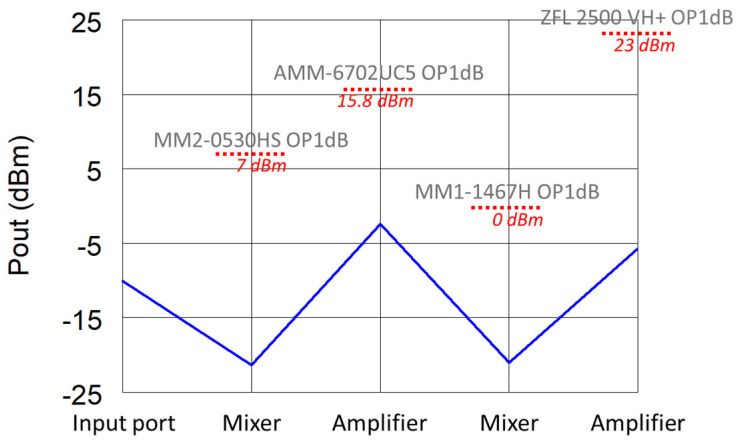
Power budget of the receiver. The input power is equal to −20 dBm.

Finally, the power budget of the receiver was simulated to verify the compliance of the components’ dynamic ranges within the entire chain and reported in Figure 7. To this purpose, the expected maximum input power of −20 dBm was considered.

## 4. Receiver Performance

**Figure 8:**
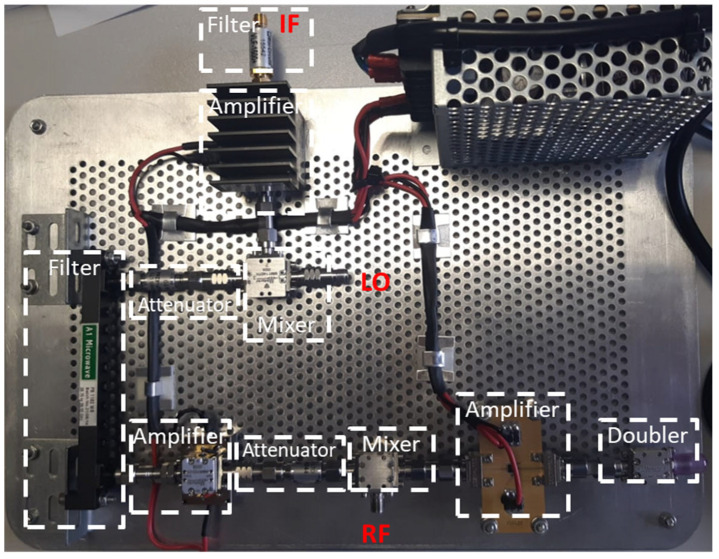
Picture of the microwave receiver.

In Figure 8, a picture of the entire receiver on a test platform is shown.

**Figure 9:**
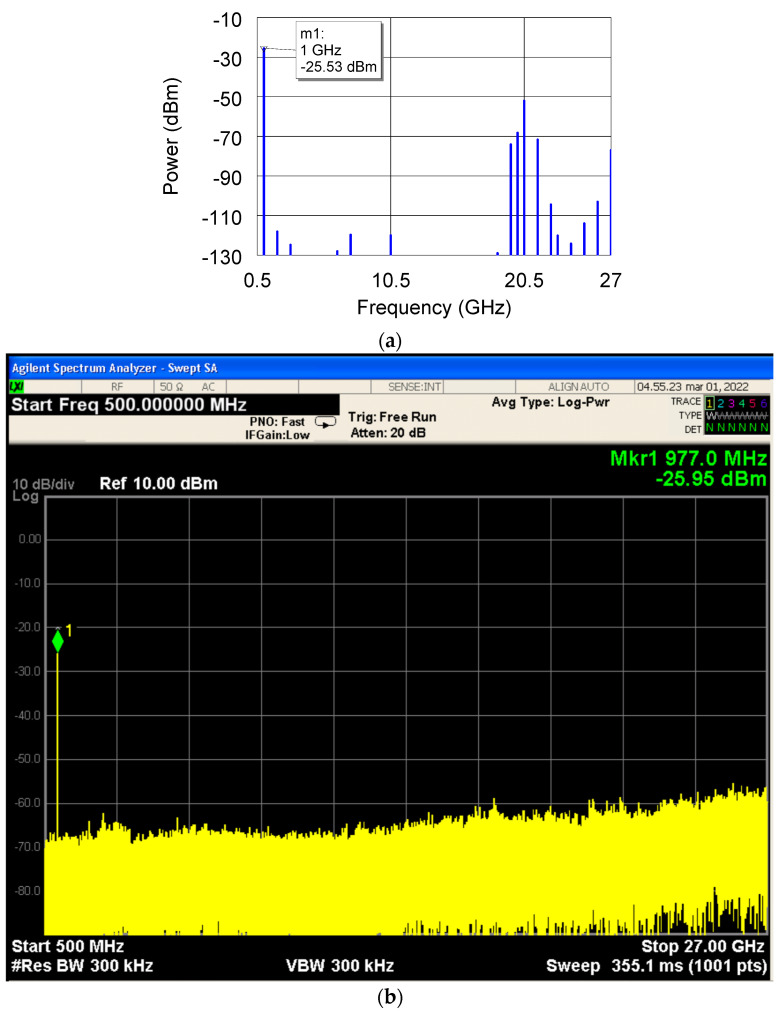
(**a**) Simulated and (**b**) measured frequency spectrum at the output of the receiver for an input signal with a center frequency and power equal to 2 GHz and −30 dBm, respectively.

The proper behavior of the receiver was tested by injecting tones within the entire frequency operating bandwidth, i.e., 2–18 GHz. In Figure 9a, the simulated output spectrum for an input power of −30 dBm at the frequency of 2 GHz is reported. The simulated performance in terms of spurious rejection is confirmed by the measured data under the same conditions, as shown in Figure 9b.

**Figure 10:**
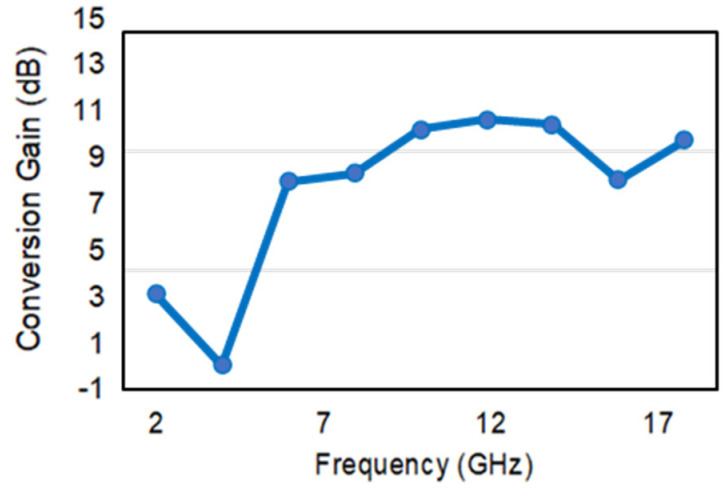
The measured conversion gain of the receiver. The input power is equal to −30 dBm.

The measured down-converter conversion gain confirms the expected behavior over the entire frequency bandwidth. It was extracted by injecting input tones ranging from 2 GHz to 18 GHz, with an input power of −30 dBm. The conversion gain is reported in Figure 10.

As anticipated in the Introduction, the measured performance of the receiver confirmed the simulated behavior. A severe reduction of both the number and level of spurious signals is evidenced in Figure 9b, where the simulated results reported in Figure 9a were confirmed. The spurious level was maintained below the minimum signal detectable by the employed spectrum analyzer. This demonstrates a spurious level suppression equal to at least −45 dBc.

This very high spurious rejection was obtained without employing expensive systems but only exploiting the available COTS components. A quite flat compression gain was obtained, particularly from 6 GHz to 18 GHz. The higher variability in the low-frequency bandwidth can be attributed to the non-flat behavior of the amplification stages, which, of course, can be mitigated in the next revisions of the project. The power consumption of the microwave section with the amplifiers switched on is lower than 1 W. Due to the rapid passage of the CubeSat footprint on the ground transmitter, the receiving systems can be switched off for almost 24 h/day and switched on for a few minutes. This very low-duty cycle allows us to keep down energy consumption.

The main performance of the downconverter is reported in Table 2.

In the scientific literature, a microwave down-converter with these characteristics is not present, thus representing a good contribution to design and development in this field, hopefully stimulating research within the community.

It is again worth highlighting the lack in the scientific literature of papers focused on very wide-band receivers. Often, commercial receivers are exploited aboard the CubeSat. As reported in Section 2, they are tailored for a narrow frequency bandwidth required by the specific application. This makes it challenging to implement an accurate performance comparison.

The idea behind this work is to invert this standard approach by adding flexibility to CubeSat communication systems to be reused for different applications. This feature can be very beneficial not only in enabling different applications without changing the hardware but also in providing the largest bandwidth if required by the ground system.

## 5. Conclusions

In this contribution, a microwave receiver is proposed as the wideband front-end for CubeSat applications. After a preliminary simulation phase devoted to the system design, the entire receiver was accurately tested and tuned. COTS components were employed with the task of designing a cost-effective system, which is one of the main requirements of CubeSat projects. The minimum spurious contribution was confirmed by an accurate experimental characterization, allowing an easy system integration for the new frontier of CubeSat technologies. In detail, the spurious level was maintained below the minimum detectable signal by demonstrating at least a −45 dBc suppression. Future research might be directed towards the development of compact-size receivers with low measured energy consumption to be finally tested on board in a real space environment.

## Figures and Tables

**Table 1:** Values of the simulated filter elements.

Element Type	Element ID	Value
Resistor	R1	5 Ω
Inductor	L1	10.92 nH
Inductor	L2	6.77 pH
Inductor	L3	18.11 nH
Inductor	L4	6.22 pH
Capacitor	C1	2.72 fF
Capacitor	C2	4.39 pF
Capacitor	C3	1.64 fF
Capacitor	C4	4.78 pF

**Table 2:** The main performance of the reported downconverter.

Frequency (GHz)	2–18
Average CG (dB)	8.4
Minimum spurious suppression level (dBc)	−45
Microwave section power consumption (W)	1

## Data Availability

Not applicable.
